# Molecular Modelling of Polychlorinated Dibenzo-p-Dioxins Non-Covalent Interactions with β and γ-Cyclodextrins

**DOI:** 10.3390/ijms241713214

**Published:** 2023-08-25

**Authors:** Maria-Cristina Ghetu, Marian Virgolici, Alina Tirsoaga, Ioana Stanculescu

**Affiliations:** 1 Faculty of Chemistry, University of Bucharest, 4-12 Regina Elisabeta Bd., 030018 Bucharest, Romania; 2 Horia Hulubei National Institute of Research and Development for Physics and Nuclear Engineering, 30 Reactorului Str., 077125 Magurele, Romania; mvirgolici@nipne.ro

**Keywords:** non-covalent interaction, molecular modelling, dioxins, cyclodextrins

## Abstract

Polychlorinated dibenzo-p-dioxins (PCDD) are persistent organic pollutants which result as byproducts in industrial or combustion processes and induce toxicity in both wildlife and humans. In this study, all seven PCDD, tetrachlorinated dibenzo-p-dioxins (TCDD), pentachlorinated dibenzo-p-dioxins (P5CDD), hexachlorinated dibenzo-p-dioxins (H6CDD), heptachlorinated dibenzo-p-dioxins (H7CDD), and octachlorinated dibenzo-p-dioxins (OCDD) were studied in interaction with two cyclodextrins, β-CD and γ-CD, resulting in a total of 40 host–guest complexes. The flexibility of the cyclodextrins was given by the number of glucose units, and the placement of the chlorine groups on the dioxins structure accounted for the different complex formed. Various geometries of interaction obtained by guided docking were studied, and the complexation and binding energy were calculated in the frame of MM+ and OPLS force fields. The results show that the recognition of the PCDD pollutants by the CD may be possible through the formation of PCDD:CD inclusion complexes. This recognition is based on the formation of Coulombic interactions between the chlorine atom of the PCDD and the primary and secondary hydroxyl groups of the CD and van der Waals interaction of the CD hydrophobic cavity with PCDD aromatic structures. Both MM+ and OPLS calculus resulted in close values for the complexation and binding energies. Molecular mechanics calculations offer a proper insight into the molecular recognition process between the PCDD compounds and CD molecules, proved by a good description of the C-H···O bonds formed between the guest and host molecules. It was shown for the first time that CD may efficiently trap PCCDs, opening the way for their tremendous potential use in environmental remediation.

## 1. Introduction

**Figure 1:**
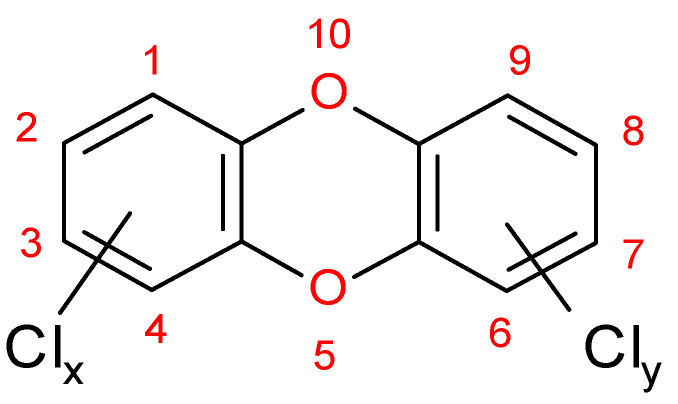
Polychlorinated dibenzo-p-dioxins (PCDD) general structure with the International Union of Pure and Applied Chemistry atom numbering scheme (1–10).

Polychlorinated dibenzo-p-dioxins (PCDD) are persistent organic pollutants, toxic for both wildlife and humans [1,2,3]. The term “dioxin” refers to polyhalogenated dibenzo-p-dioxins (Figure 1) and dibenzofurans, which result as byproducts in industrial or combustion processes when a chlorine source is present [4]. Besides the anthropogenic production of the dioxins, natural events (volcano eruptions or wildfires) can generate limited quantities of PCDDs derivatives [5]. In total, there are seven PCDD compounds. Each compound exhibits variable toxic potency, but the action mechanism is the same. Therefore, a relative potency ranking was created in order to assign a toxic equivalency factor (TEF) to each compound, as compared to the most toxic dioxin, 2,3,7,8-tetrachlorinated dibenzo-p-dioxin (2,3,7,8-TCDD), which has a TEF equal to 1 [6].

PCDDs are formed as undesirable byproducts in all combustion processes. They are also formed during some industrial processes, such as the production of pesticides and paper or cellulose, and in the iron and steel industry and non-ferrous metallurgy [7]. Dioxins are not only pollutants, but they are also highly toxic for humans and can cause serious health problems [8] including increasing blood lipids, immune inflammatory disorders, organ system dysfunction, mental disability, and cancer, but mainly affect the endocrine system by disrupting its functions by activating the aryl hydrocarbon receptor (AhR) signaling pathways [9].

The AhR is most often called the “dioxin receptor”. AhR is a nuclear transcription factor that regulates various physiological and developmental processes of different organs. It is localized in the cytoplasm, and it is activated by high-affinity lipophilic xenobiotic compounds, as well as low-affinity endogenous ligands [10] that diffuse through the plasma membrane. The activation of this receptor induces a broad spectrum of biochemical and toxic effects [11]. Most if not all toxic and biological effects of these compounds are mediated through the aryl hydrocarbon receptor (AhR), which is a cytosolic receptor protein present in most vertebrates. The AhR has a high affinity for 2,3,7,8-TCDD compound due to the increasing chlorination on the lateral sites of the molecule [12].

Cyclodextrins are a family of cyclic oligosaccharides with a hydrophilic surface and a lipophilic central cavity [13]. Due to their cone-shape structure, they have the ability to solubilize and stabilize guest compounds. Cyclodextrins are formed in nature by the digestion of cellulose by bacteria and are composed of varying number of glucose units held together by α-1,4 glycosidic bonds. The naturally occurring varieties contain six, seven, or eight glucose units. Cyclodextrins with more than eight units are less common in nature, and the ones with five units are only synthetic [14].

The main application of CDs is as complexing agents. Inclusion complexes are formed based on the “guest–host” interaction, where the CD’s cavity acts as host for a molecule. The outer sphere of CD is hydrophilic, while its cavity is hydrophobic. A molecular dynamics simulation revealed that the hydrophobicity dominates inside the cavity of hydrated β-CD (where an ordered configuration of water molecules was put in evidence to interact only with each other by forming tightly packed hydrogen bonding networks which is surrounded by non-polar surface of the inner walls of the CD), whereas at the top and bottom of cone-shape structure of CD, interactions with water are mostly hydrophilic in nature [15]. This means that the cavity can act as a host for compounds with poor solubility in polar solvents by providing a hydrophobic inner micro-environment, and that the exterior of CD is compatible with water, which allows hydrogen bonding cohesive interaction [16].

This propriety of CDs to form inclusion complexes is used in their applications. Due to their structure, CDs are able to enhance the solubility of many poorly soluble compounds by forming a complex with their functional groups. These types of complexations can also increase the stability of unstable compounds, because when a molecule in entrapped within its cavity, it is difficult for a reactant to diffuse into the cavity and react with the protected guest. Moreover, molecules that cause unpleasant smells or tastes can be hidden from the sensory receptors by entrapment within the CD’s cavity [17].

Molecular dynamics simulations performed on the α-naphthalen-acetic acid/methylated β-CD complex revealed the hydrophobic character of the CD cavity due to the favored binding mode of the guest, which has its naphthyl group accommodated inside of the host cavity and its carboxymethyl groups pointed towards the primary host rim—all the inclusion complexes were stabilized by the hydrogen bonds formed with the glycosidic or secondary methoxy oxygen atoms of the host [18].

The main driving force for the spontaneous entrance of PCB52 (2,2′,5,5′–tetrachlorobiphenyl) into the cavity of CD molecules is represented by electrostatic and van der Waals interactions between the host and guest molecules as was proved by both theoretical modeling (DFT calculations and MD simulations) and FTIR and Raman spectroscopy measurements [19] which is also in good agreement with our results.

The host–guest inclusion complexes of CD with different guest molecules are explored by various analytical methods such as UV-Vis, fluorescence, circular dichroism, FTIR, Raman spectroscopy, NOE, ROESY, XRD, microscopies, and calorimetric techniques [20]. Thus, due to experimental limitations, molecular modelling represents a useful tool to study the non-covalent interactions involving very toxic and persistent organic pollutants and CDs. This is also the case for adsorption of 2,3,7,8-tetrachlorinated dibenzo-p-dioxins compound on both Ti (N, Ag) doped graphene [21] and Al-doped carbon nanotubes [22] which was investigated by theoretical calculations due to the strong toxicity of this compound [23] in order to evaluate new bioremediation technologies [24]. These results show that a charge transfer between the TCDD molecule and metallic atoms (Ti, Al) is responsible for the stabilization of the guest molecule on the surface of doped carbon substrate due to an orbital mixing near the Fermi level [21,22].

In this study we used seven different PCDD compounds, tetrachlorinated dibenzo-p-dioxins (TCDD), pentachlorinated dibenzo-p-dioxins (PCDD), hexachlorinated dibenzo-p-dioxins (H_6_CDD), heptachlorinated dibenzo-p-dioxins (H_7_CDD), and octachlorinated dibenzo-p-dioxins (OCDD), the 2,3,7,8 chlorine substituted congeners, known as the most toxic of the total 135 PCDD structures. All those seven compounds were studied interacting with two cyclodextrins, β-CD and γ-CD. The novelty of the study consists of showing for the first time by rapid molecular mechanics calculations that both CDs are capable of including the PCDD molecules. To our knowledge there is only a paper of 2011, describing by laborious quantum mechanics and molecular dynamics calculation the inclusion of TCDD by beta-cyclodextrin [25]. In our case, the MM calculations provide a reliable, rapid, and cheaper approach which is able to point out a spontaneous interaction between a large series of dioxins derivatives and CD molecules. The results could be of interest for environmental remediation applications in capturing PCDD on CD substrate and also to analytical chemistry for chromatographic identification of the PCCDs.

## 2. Results and Discussion

For our study, we analyzed different inclusion complexes because different numbers of chlorine atoms affect the stability of the complex, given the fact that this creates a difference in Keesom and London electrostatic forces [26].

**Figure 2:**
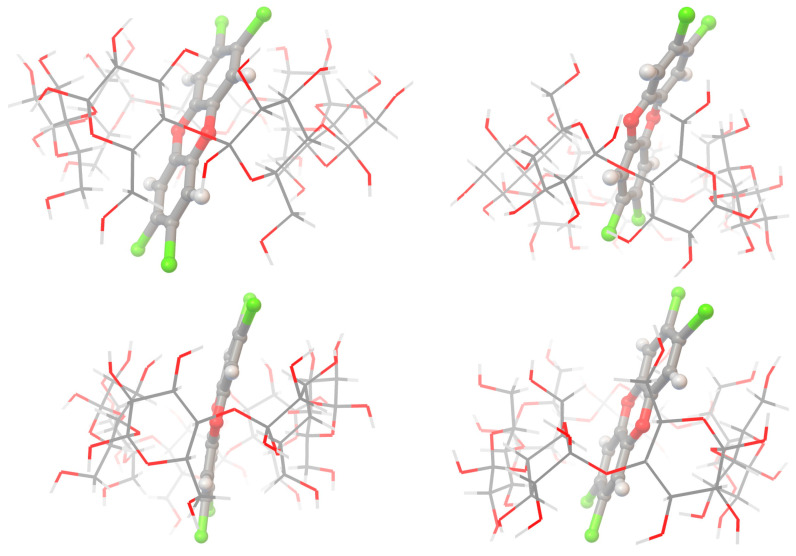
An example of the inclusion complexes obtained by MM+ calculation (top structures) OPLS (bottom structures) for: TCDD2378: beta CD complexes visualized with the Python Molecule Viewer [27]. (color code: carbon (dark gray), chlorine (green), oxygen (red), hydrogen atoms (gray)).

In the case of the formed complexes, the PCDDs are placed at a distance from the center of the CD that is favorable for forming Coulombic interactions between the chlorine atom of the PCDD, and primary or secondary hydroxyl groups of the CD (see Figure 2). The geometry optimization was realized both using MM+ (Table 1 and Table 2), and OPLS (Table 3 and Table 4) methods.

The complexation energy and the binding energy with all their components were calculated for all of the 40 inclusion complexes (see Figure 2 and Tables S1–S4 of Supplementary Materials). As expected, the van der Waals energy (E_vdW_) has the biggest weighted value (percentage); therefore, it has the most important contribution, and represents the non-covalent interactions within the inclusion complex as found by Pan et al. [25]. In our calculations, the electrostatic interactions and hydrogen bonding seem to have a smaller contribution to the binding energy compared to the vdW ones maybe due to their mathematical description as dipole–dipole interactions.

Table 1, Table 2, Table 3 and Table 4 report the results for all inclusion complexes studied and the energy variation is plotted in Figures S1–S4 of Supplementary Materials, in order to provide a better visualization. Compared with the E_vdW_, all of the other terms have a lower contribution, and thus are not presented. The results follow a common trend, where the induction and dispersion forces which account for van der Waals interactions play an important role in the formation of inclusion complexes between hydrophobic CDs cavity and guest molecules, even if the exchange of interactions during hydration/dehydration of the host was not taken into consideration [28]. Moreover, the hydrophobic interactions might be considered as a very important driving force for this molecular recognition process, since halogen atoms are typically described as hydrophobic residues and the presence of chlorine atoms in a molecule increases its lipophilicity and consequently its hydrophobicity.

Taking into consideration the classical description of the halogen atoms, featuring pronounced electronegativity values, they present an important -*I* inductive electron withdrawing effect when attached to carbon atoms, becoming thus negatively polarized (δ-) sites. However, a closer look at the charge distribution around the halogen atom proves them to be not uniform; based on a new concept introduced by Kollman’s studies on diatomic molecules [29] and following a theoretical calculation on halogenated methanes, Brinck et al. [30] pointed out that the electron density around a covalently bonded halogen atom has an anisotropic distribution, thus: (i) as expected, orthogonal to the covalent-bond sides of the halogen atom is a higher electron density where the electrostatic potential is negative, and (ii) on the outer side of the halogen atom, along the extension of the covalent-bond, a depleted electron density region (named σ-hole) was found being associated to a positive electrostatic potential. Therefore, in crystalline packed structures, the halogen atoms might have favorable interactions both with nucleophiles placed along their sigma-bonds and with electrophiles from lateral directions [30]. The existence of halogen bonds was demonstrated not only in organic chemical systems, but also in crystals of coordination compounds [31,32,33,34,35,36].

A halogen bond (XB) is thus an electrostatically attractive intermolecular non-covalent interaction which occurs between an electrophilic region around a halogen atom and a nucleophilic region of a donor atom associated with a Lewis base which is accessible to the sigma-hole [37,38]. Moreover, the XB strength depends on some specific features, such as: (i) tunability, (ii) directionality, (iii) nature of the donor of the halogen-bond, and (iv) the distance between the halogen atom (X) and donor atom (D). The tunability of the XB can be modulated if an electron-withdrawing substituent is bound in the vicinity of the halogen atom, and thereby the size of the σ-hole increases with the polarizability of the heaviest halogen atom and decreases with its electronegativity. The well-known directionality of the XB relies upon the necessity of a linear arrangement between the halogen and the donor atoms since deviations of up to 40° from the linearity induce a weaker halogen bond because in such way the σ-hole would be less accessible to the electron donor. Even for the strong Lewis bases (i.e., oxygen from carbonyl and nitrogen from amide moieties) the separation between the atoms involved in XB must be smaller than the sum of their van der Waals radii, in order to induce a significant attractive non-covalent interaction [39,40]. XBs obviously rely on hydrophobic environment features, in contrast with the hydrogen bonds [41] but, although our polychlorinated-containing derivatives exhibit a high binding affinity to the hydrophobic CDs cavity, the driving forces for the formation of inclusion complexes are unlikely to be of halogen bonding type due to the highly directional nature of XBs.

The shape of the molecular electrostatic potential distribution modelled for some PCDDs [42] exhibits an effective localization of electron-rich regions on halogen atoms, whereas the negative potential values on oxygen atoms are closer to neutrality than expected, due to the strong electron-withdrawing inductive strength of the chlorine atoms; the competition for the polarizable electronic charge induced by the distribution of the chlorine atoms in the molecule might also be considered as having some contribution during the molecular recognition process. Consequently, above the benzo-rings of PCDDs structures the existence of a positive potential was reported [42,43], so that the guest-molecule exhibits alternating regions with negative and positive potentials. Thus, the non-covalent bonding involved in the complexation process might be interpreted in terms of long-range Coulombic interactions, since the theoretical description of charge distribution involves polarization phenomena and the related dispersion contributions [44].

It is observed that there is a value difference between the inclusion complexes formed with β-CD and those formed with γ-CD. This difference may be attributed to the higher flexibility of the γ-CD, which may facilitate the inclusion of the PCDDs molecules. Molecular dynamics studies pointed out that hydrophobic cavities of β- and γ-CDs are more polarizable compared to α-CD ones, and therefore the non-polar part of the guest molecule is attracted inside the β- and γ-cyclodextrin structures [45]. Moreover, a mutual polarization between the PCDDs molecules and the CDs may also contribute to the stabilization of non-covalent interactions, which are involved in the complexation process [46].

From the small energy difference of the complexes formed by Coulombic interactions of the chlorine atom and the primary and secondary hydroxyl groups, it may result that the PCDD does not have a preference for one type of hydroxyl group of the CD. However, some weak electrostatic interactions were described as host–guest C-H···O bonds when the (H···O) distance is less than 3Å and the angle (C-H···O) is more than 90° as presented in the previous literature reports [47,48,49]. Regardless of the docking mode, four C-H···O bonds are formed in TCDD2378: beta CD complexes, such as between the H5 and H6 pyranosidic protons from the inner surface of the CD and O atom from the guest molecule and also between glycosidic O atom and H atom linked to an aromatic carbon of the PCDD compounds. This result is in reasonable agreement with the previous reported data [25], when the DM simulations and QM calculations with a higher theoretical level were performed to investigate the inclusion complexation of TCDD with β-CD. Regarding the series of PCDD derivatives investigated in this work, the number of the C-H···O bonds formed during the inclusion complexes process depend on the number of the chlorine atoms presented in the guest molecule.

Furthermore, the small differences obtained for binding and interaction energies using MM+ and OPLS indicate an adequate approach of the PCDD:cyclodextrin interaction calculation.

## 3. Materials and Methods

The simulation of interaction between the seven PCDD compounds and the two CDs was conducted using molecular mechanics (MM+) and optimized potential for liquid simulations (OPLS) force fields, both included in the Hyperchem program v.6.01 [50]. The starting geometry of CDs was obtained from the Protein Data Bank using the X-ray structures 1VFO for β-CD and 1D3C for γ-CD [51].

For an accurate simulation, MM+ calculations were performed for structure optimization using the Polack–Ribiere algorithm, with a gradient of 0.01 kcal/mol Å. In the case of OPLS calculations, the dielectric constant was kept constant, and the scale factor used was 10, both in order to account for the solvent effect.

The isolated structures of each CDs and PCDDs were optimized without imposing any symmetry restrictions. The inclusion complexes were obtained using each PCDD compound interacting with each CD. In order to study the influence of the number of chlorine atoms, different starting geometries were used. Geometry optimizations were performed for 7 PCDDs, 2 CDs, and 40 inclusion complexes. The geometries of the inclusion complex were visualized with the Python Molecule Viewer v1.5.7p1 as implemented in MGL Tools v.1.5.7 tarball installer [27].

**Figure 3:**
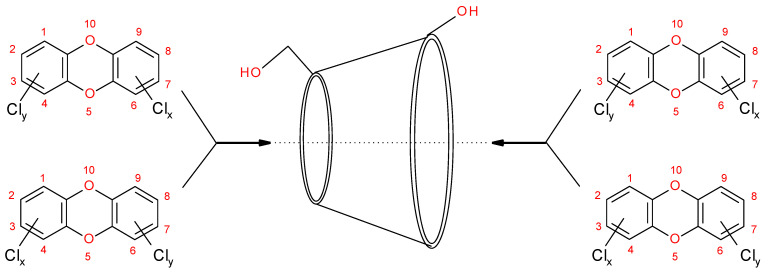
Possible inclusion modes for PCDDs complex formation by approaching the primary, respectively the secondary hydroxyl groups of the beta and gamma CDs. International Union of Pure and Applied Chemistry atom numbering scheme (1–10) of PCCD is given. CDs cavity central axis is represented with dotted line and the PCDDs alternative CD’s approach direction with arrows.

In order to identify the energetically favorable complexation structures of PCDDs derivatives with each CDs in a 1:1 ratio, a molecular docking approach was used. The inclusion complexes were obtained by placing the PCDD parallel respective to the cavity opening central axis of the CD. The guest molecule was placed on the central direction of the CD’s cavity, with an inter-molecular distance of at least 3 Å, but not exceeding 6 Å, according to a docking procedure proposed by Camacho and collaborators [52]. The interaction study was performed for two different binding orientation of the PCDDs derivatives in which chlorine atoms of the guest molecule is either near the primary hydroxyl side groups (O6 side) of the CD or near the secondary ones (O2/O3 side) as depicted in Figure 3.

**Scheme 1:**
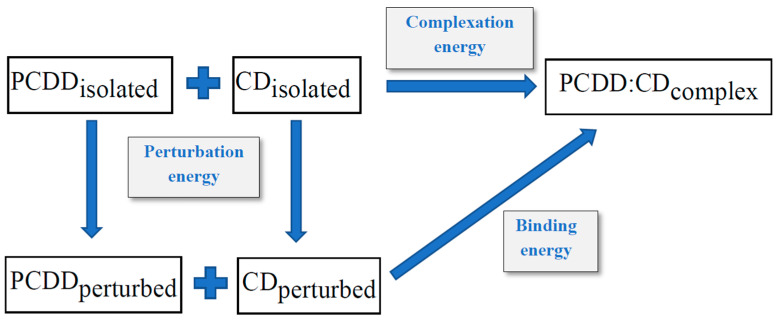
Host–guest interaction energy calculation for the PCDD:CD complexes.

The host–guest interaction energy calculation is represented in Scheme 1 [26,53,54,55,56], as follows:

By applying general principles of thermodynamics on Scheme 1, we have used the following relations [57], Equations (1) and (2), for the complexation energy and for the binding energy, respectively from the total energy (E):

ΔE_complexation_ = (E_PCDDisolated_ + E_CDisolated_) − E_PCDD:CD_ (1)

ΔE_binding_ = (E_PCDDperturbed_ + E_CDperturbed_) − E_PCDD:CD_ (2)

Binding energies and complexation energies have positive values for favorable interactions [57].

In the molecular mechanics force fields, the total energy of the system (Equation (3)), is composed of several terms as described in [50]:

E_total_ = E_bond_ + E_angle_ + E_dihedral_ + E_vdW_ + E_Hbond_ + E_electrostatic_ (3)

The first three terms describe bonding interactions of covalent-linked atoms, while the last three ones describe the non-covalent contributions. The magnitude of van der Waals interactions is estimated on the basis of Lennard-Jones (L-J) 6–12 potential function, the H-bond term is parameterized as an L-J 10–12 type potential function, while the electrostatic interactions are computed with Coulomb’s law. Molecular mechanics approach was chosen not only due to the decrease in the computational cost, but because it is also suitable for the conformational studies and interaction energy calculation as well [58]. On one hand, molecular mechanics is not appropriate for simulate any chemical process where the understanding of electronic structure is explicitly required, but on the other hand, in agreement with some experimental findings, the molecular geometries are well modeled by using different analytical functions which describe classical interactions potentials between bonded and non-bonded atoms. Empirical force fields avoid solving Schrodinger’s equation by using the expression of the potential energies as a function of the nuclear coordinates only, where the dependence upon electrons is implicitly treated by suitable parametrization [59].

## 4. Conclusions

In conclusion, this study presented that, from molecular modelling calculations, the recognition of the PCDD pollutants by the CD may be possible through the formation of PCDD:CD inclusion complexes. This molecular recognition and thus the stability of these host–guest complexes is due to the Coulombic interactions between the chlorine atom of the PCDD and the primary and secondary hydroxyl groups of the CD, as well as van der Waals/hydrophobic interactions between the CD cavity and aromatic nuclei of PCCDs derivatives. Both MM+ and OPLS calculus resulted in close values for the complexation and binding energies. This work may be useful for further research by computational methods, in developing gas chromatographic separation of the named pollutants as well as for environmental applications of PCDD trapping systems based on CD. However, the current work proves that it is reliable to use a molecular mechanic approach to model the non-covalent interactions involving molecular recognition process between cyclodextrins and dioxins derivatives, since weak electrostatic interactions as C-H···O bonds were well depicted following MM methods.

## Figures and Tables

**Table 1:** Energy of the inclusion complex of the perturbed state and their vdW components formed by approaching the secondary hydroxyl groups of β-CD (1) and γ-CD (2) obtained by MM+ geometry optimization.

MM+	E_complex_ (1)	E_vdWcomplex_ (1)	E_perturbed_ (1)	E_vdWperturbed_ (1)	E_complex_ (2)	E_vdWcomplex_ (2)	E_perturbed_ (2)	E_vdWperturbed_ (2)
TCDD_2378_	52.30	9.21	77.63	34.69	70.96	16.27	107.05	46.03
PCDD_12378_-123	58.43	10.12	82.79	34.35	76.29	24.11	95.08	42.33
PCDD_12378_-78	55.45	7.90	84.04	35.25	72.09	20.04	96.95	42.41
H_6_CDD_123478_-1234	65.27	16.84	88.07	36.74	78.16	23.64	99.43	43.89
H_6_CDD_123478_-78	63.77	13.90	87.65	37.60	77.10	21.25	99.71	43.16
H_6_CDD_123678_	63.80	15.10	91.05	40.99	81.29	22.55	108.66	48.22
H_6_CDD_123789_	65.79	16.38	92.52	41.63	81.46	24.61	107.58	48.36
H_7_CDD_1234678_-1234	66.58	16.77	87.98	37.25	79.74	23.51	103.61	44.80
H_7_CDD_1234678_-678	61.58	10.21	88.25	36.38	79.08	24.54	100.77	44.92
OCDD_12346789_	69.48	17.44	91.59	38.43	82.16	24.48	107.05	46.03

**Table 2:** Energy of the inclusion complex of the perturbed state and their vdW components formed by approaching the primary hydroxyl groups of β-CD (1) and γ-CD (2) obtained by MM+ geometry optimization.

MM+	E_complex_ (1)	E_vdWcomplex_ (1)	E_perturbed_ (1)	E_vdWperturbed_ (1)	E_complex_ (2)	E_vdWcomplex_ (2)	E_perturbed_ (2)	E_vdWperturbed_ (2)
TCDD_2378_	54.04	11.07	77.98	34.39	76.40	24.75	93.43	40.70
PCDD_12378_-123	58.66	15.88	80.37	35.51	72.15	18.61	90.11	34.21
PCDD_12378_-78	55.33	10.29	81.59	35.02	74.94	25.27	95.63	42.38
H_6_CDD_123478_-1234	64.47	18.87	84.53	35.60	80.69	27.01	100.38	43.54
H_6_CDD_123478_-78	57.79	10.64	84.90	36.80	81.95	27.50	99.99	43.45
H_6_CDD_123678_	69.16	18.90	92.32	41.50	84.23	27.02	107.46	48.54
H_6_CDD_123789_	74.42	27.62	90.60	41.75	80.21	23.61	103.20	43.73
H_7_CDD_1234678_-1234	66.17	17.00	88.74	38.03	79.55	24.34	102.83	44.74
H_7_CDD_1234678_-678	64.32	13.89	90.18	37.77	79.97	23.30	104.23	44.82
OCDD_12346789_	69.04	16.93	93.03	39.02	80.56	23.31	104.23	40.82

**Table 3:** Energy of the inclusion complex of the perturbed state and their vdW components formed by approaching the secondary hydroxyl groups of β-CD (1) and γ-CD (2) obtained by OPLS geometry optimization.

OPLS	E_complex_ (1)	E_vdWcomplex_ (1)	E_perturbed_ (1)	E_vdWperturbed_ (1)	E_complex_ (2)	E_vdWcomplex_ (2)	E_perturbed_ (2)	E_vdWperturbed_ (2)
TCDD_2378_	52.72	−41.72	85.95	−8.49	77.97	−32.22	100.39	−9.80
PCDD_12378_-123	58.95	−37.93	87.18	−9.69	76.95	−33.64	100.46	−10.13
PCDD_12378_-78	57.34	−38.31	86.87	−8.78	74.63	−37.65	103.77	−8.51
H_6_CDD_123478_-1234	59.19	−37.29	87.20	−9.39	74.43	−35.77	103.38	−6.82
H_6_CDD_123478_-78	56.33	−39.99	86.96	−9.39	76.36	−36.89	107.98	−5.27
H_6_CDD_123678_	53.50	−43.28	87.62	−9.15	73.72	−36.66	101.55	−8.83
H_6_CDD_123789_	52.50	−43.02	86.38	−9.15	74.85	−34.63	105.06	−4.42
H_7_CDD_1234678_-1234	56.78	−41.52	88.17	−10.13	75.83	−37.60	105.20	−8.23
H_7_CDD_1234678_-678	52.71	−44.75	87.87	−9.59	73.28	−37.60	101.88	−9.01
OCDD_12346789_	56.96	−42.90	89.10	−10.77	72.39	−37.64	105.19	−4.84

**Table 4:** Energy of the inclusion complex of the perturbed state and their vdW components formed by approaching the primary hydroxyl groups of β-CD (1) and γ-CD (2) obtained by OPLS geometry optimization.

OPLS	E_complex_ (1)	E_vdWcomplex_ (1)	E_perturbed_ (1)	E_vdWperturbed_ (1)	E_complex_ (2)	E_vdWcomplex_ (2)	E_perturbed_ (2)	E_vdWperturbed_ (2)
TCDD_2378_	56.07	−39.07	86.67	−8.47	81.46	−27.05	100.29	−8.22
PCDD_12378_-123	57.97	−38.71	87.36	−9.33	80.82	−30.40	101.85	−9.37
PCDD_12378_-78	52.58	−40.34	84.66	−8.26	74.70	−32.03	102.61	−4.11
H_6_CDD_123478_-1234	59.18	−11.31	84.77	−13.66	80.36	−29.02	104.26	−5.12
H_6_CDD_123478_-78	56.33	−39.99	85.66	−9.17	55.41	−39.42	85.66	−9.17
H_6_CDD_123678_	60.61	−33.65	85.03	−9.22	76.10	−33.67	104.20	−5.57
H_6_CDD_123789_	57.38	−38.27	86.25	−9.41	82.23	−27.11	104.68	−4.66
H_7_CDD_1234678_-1234	61.63	−32.62	85.05	−9.19	78.77	−30.61	100.61	−8.77
H_7_CDD_1234678_-678	58.81	−37.93	86.96	−9.77	82.36	−27.65	105.04	−4.97
OCDD_12346789_	61.42	−33.35	85.17	−9.60	78.81	−34.37	96.69	−16.49

## Data Availability

Data are available on request from the authors.

## Supplementary Materials

The following supporting information can be downloaded at: https://www.mdpi.com/article/10.3390/ijms241713214/s1.
