# Supplementary material for: Molecular Modelling of Polychlorinated Dibenzo-p-Dioxins Non-Covalent Interactions with β and γ-Cyclodextrins
Source: Int J Mol Sci. 2023 Aug 25;24(17):13214. doi: 10.3390/ijms241713214 (PMC10488135; doi:10.3390/ijms241713214)
Supplement: Supplementary file 1 [file ijms-24-13214-s001.zip › ijms-2526596-supplementary.pdf]

Supplementary Material

for

Molecular Modelling of Polychlorinated Dibenzo-p-Dioxins Non-Covalent Interactions with β and γ-Cyclodextrins

***Maria-Cristina Ghetu* [1,2], *Marian Virgolici* [2], *Alina Tirsoaga* [1*] and *Ioana Stanculescu* [1,2*]**

1. *Faculty of Chemistry, University of Bucharest, 4-12 Regina Elisabeta Bd., 030018 Bucharest, Romania; maria.cristina.ghetu@gmail.com*
2. *Horia Hulubei National Institute of Research and Development for Physics and Nuclear Engineering, 30 Reactorului Str., 077125 Magurele, Romania; mvirgolici@nipne.ro*

** Correspondence: alina.jurca@unibuc.ro (A.T.); istanculescu@nipne.ro (I.S.)*

Table S1. Complexation energy and binding energy for the inclusion complexes formed by the PCDDs with the secondary hydroxyl groups of β-CD (1), and γ-CD (2) by MM+ geometry optimization.

MM+	$\Delta E_{complexation}$ (1) (kcal/mol)	$\Delta E_{binding}$ (1) (kcal/mol)	$\Delta E_{complexation}$ (2) (kcal/mol)	$\Delta E_{binding}$ (2) (kcal/mol)
**$TCDD_{2378}$**	24.71	25.29	21.43	36.75
**$PCDD_{12378}$-123**	21.38	24.46	18.91	21.90
**$PCDD_{12378}$-78**	24.36	27.74	23.10	24.55
**H_6CDD_{123478} -1234**	17.97	23.05	20.46	23.33
**H_6CDD_{123478}-78**	19.47	24.98	21.52	24.06
**H_6CDD_{123678}**	25.55	29.23	23.45	32.34
**H_6CDD_{123789}**	23.56	28.05	23.28	29.48
**$H_7CDD_{1234678}$-1234**	19.56	21.11	21.79	25.20
**$H_7CDD_{1234678}$-678**	24.56	26.08	22.44	21.02
**$OCDD_{12346789}$**	20.17	22.11	22.89	26.79

Table S2. Complexation energy and binding energy for the inclusion complexes formed by the PCDDs with the primary hydroxyl groups of β-CD (1), and γ-CD (2) by MM+ geometry optimization.

MM+	$\Delta E_{complexation}$ (1) (kcal/mol)	$\Delta E_{binding}$ (1) (kcal/mol)	$\Delta E_{complexation}$ (2) (kcal/mol)	$\Delta E_{binding}$ (2) (kcal/mol)
**$TCDD_{2378}$**	22.97	23.95	15.99	17.03
**$PCDD_{12378}$-123**	21.15	21.71	23.05	17.96
**$PCDD_{12378}$-78**	24.48	26.26	20.26	20.69
**H_6CDD_{123478}-1234**	18.77	20.07	17.93	19.69
**H_6CDD_{123478}-78**	25.45	27.12	16.67	18.04
**H_6CDD_{123678}**	20.19	23.16	20.51	23.23
**H_6CDD_{123789}**	14.93	16.18	24.53	23.00
**$H_7CDD_{1234678}$-1234**	19.97	22.58	21.98	23.28
**$H_7CDD_{1234678}$-678**	21.82	25.86	21.56	24.26
**$OCDD_{12346789}$**	20.62	23.99	24.53	23.67

Table S3. Complexation energy and binding energy for the inclusion complexes formed by the PCDDs with the secondary hydroxyl groups of β-CD (1), and γ-CD (2) by OPLS geometry optimization.

**OPLS**	**$\Delta E_{complexation}$ (1)** (kcal/mol)	**$\Delta E_{binding}$ (1)** (kcal/mol)	**$\Delta E_{complexation}$ (2)** (kcal/mol)	**$\Delta E_{binding}$ (2)** (kcal/mol)
**$TCDD_{2378}$**	24.29	33.23	14.42	22.42
**$PCDD_{12378}$-123**	20.86	28.23	18.25	23.51
**$PCDD_{12378}$-78**	22.47	29.53	20.56	29.13
**H_6CDD_{123478}-1234**	24.05	28.01	24.19	28.95
**H_6CDD_{123478}-78**	26.91	30.63	22.26	31.62
**H_6CDD_{123678}**	35.86	34.12	31.01	27.83
**H_6CDD_{123789}**	36.85	33.87	29.88	30.21
**$H_7CDD_{1234678}$-1234**	29.36	31.39	25.70	29.37
**$H_7CDD_{1234678}$-678**	33.43	35.16	28.24	28.59
**$OCDD_{12346789}$**	32.70	32.14	32.65	32.80

Table S4. Complexation energy and binding energy for the inclusion complexes formed by the PCDDs with the primary hydroxyl groups of β-CD (1), and γ-CD (2) by OPLS geometry optimization.

**OPLS**	**$\Delta E_{complexation}$(1)** (kcal/mol)	**$\Delta E_{binding}$(1)** (kcal/mol)	**$\Delta E_{complexation}$(2)** (kcal/mol)	**$\Delta E_{binding}$(2)** (kcal/mol)
**$TCDD_{2378}$**	24.29	30.60	14.42	18.83
**$PCDD_{12378}$-123**	20.86	29.39	18.25	21.03
**$PCDD_{12378}$-78**	22.47	32.08	20.56	27.91
**H_6CDD_{123478}-1234**	24.05	25.59	24.19	23.90
**H_6CDD_{123478}-78**	26.91	29.33	22.26	30.25
**H_6CDD_{123678}**	35.86	24.42	31.01	28.10
**H_6CDD_{123789}**	36.85	28.86	29.88	22.45
**$H_7CDD_{1234678}$-1234**	29.36	23.42	25.70	21.84
**$H_7CDD_{1234678}$-678**	33.43	28.16	28.24	22.68
**$OCDD_{12346789}$**	32.70	23.74	32.65	17.88

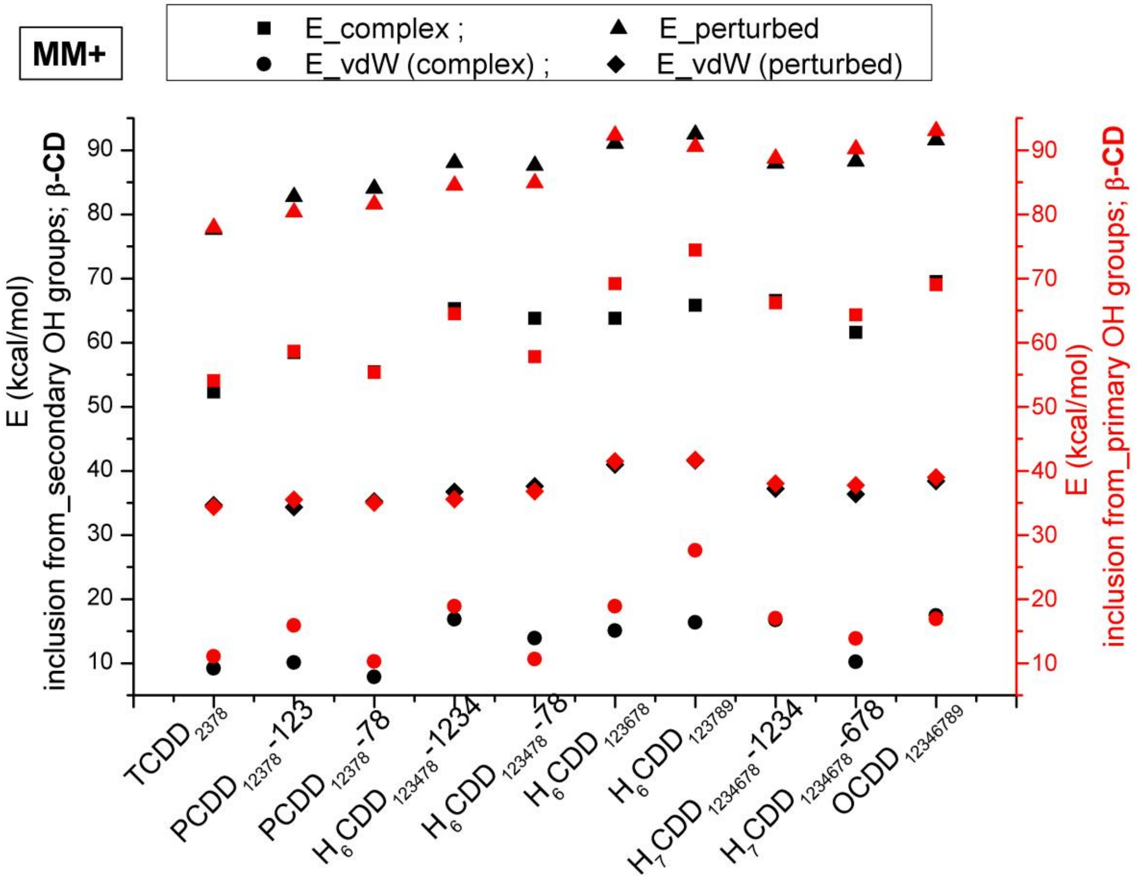

Figure S1. Complex and perturbed state energy (kcal/mol) and theirs vdW components for the inclusion complexes formed by the PCDDs approaching the secondary hydroxyl groups (black color) and the primary hydroxyl groups (red color) of β-CD by MM+ geometry optimization.

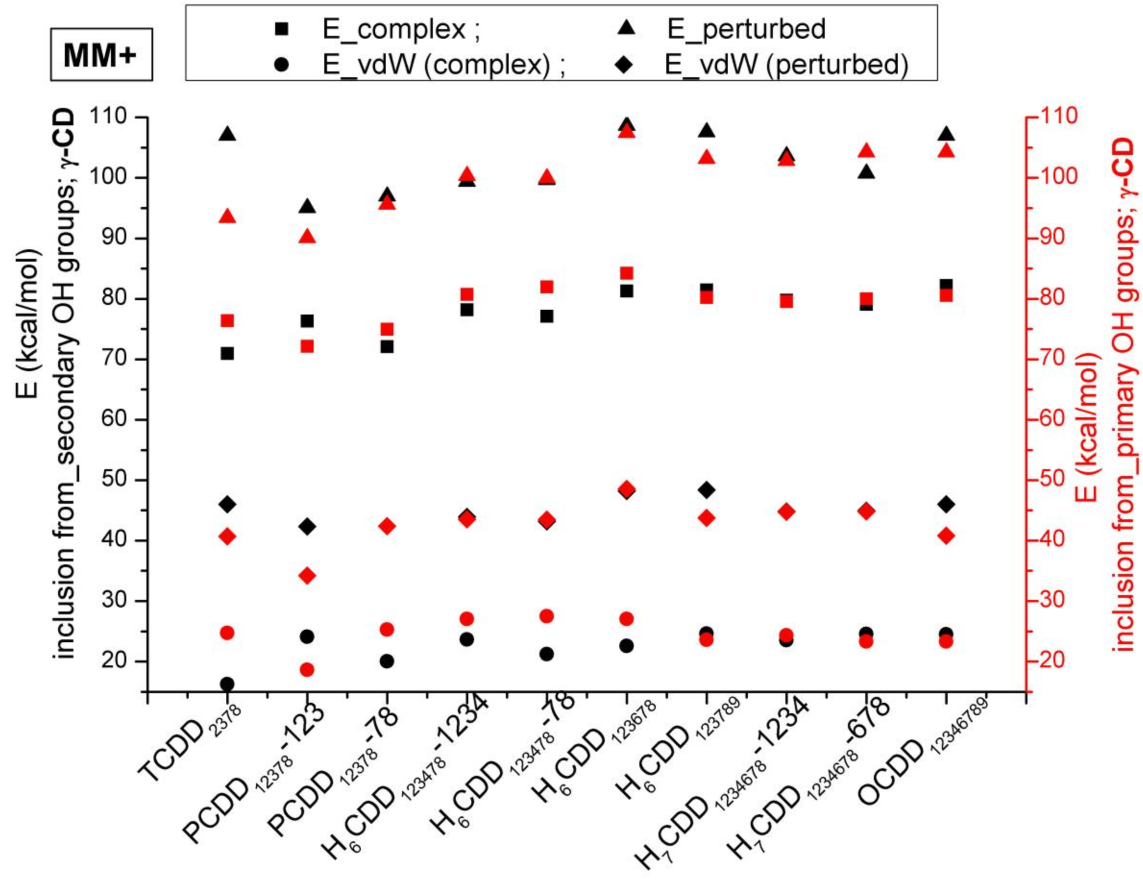

Figure S2. Complex and perturbed state energy (kcal/mol) and theirs vdW components for the inclusion complexes formed by the PCDDs approaching the secondary hydroxyl groups (black color) and the primary hydroxyl groups (red color) of γ-CD by MM+ geometry optimization.

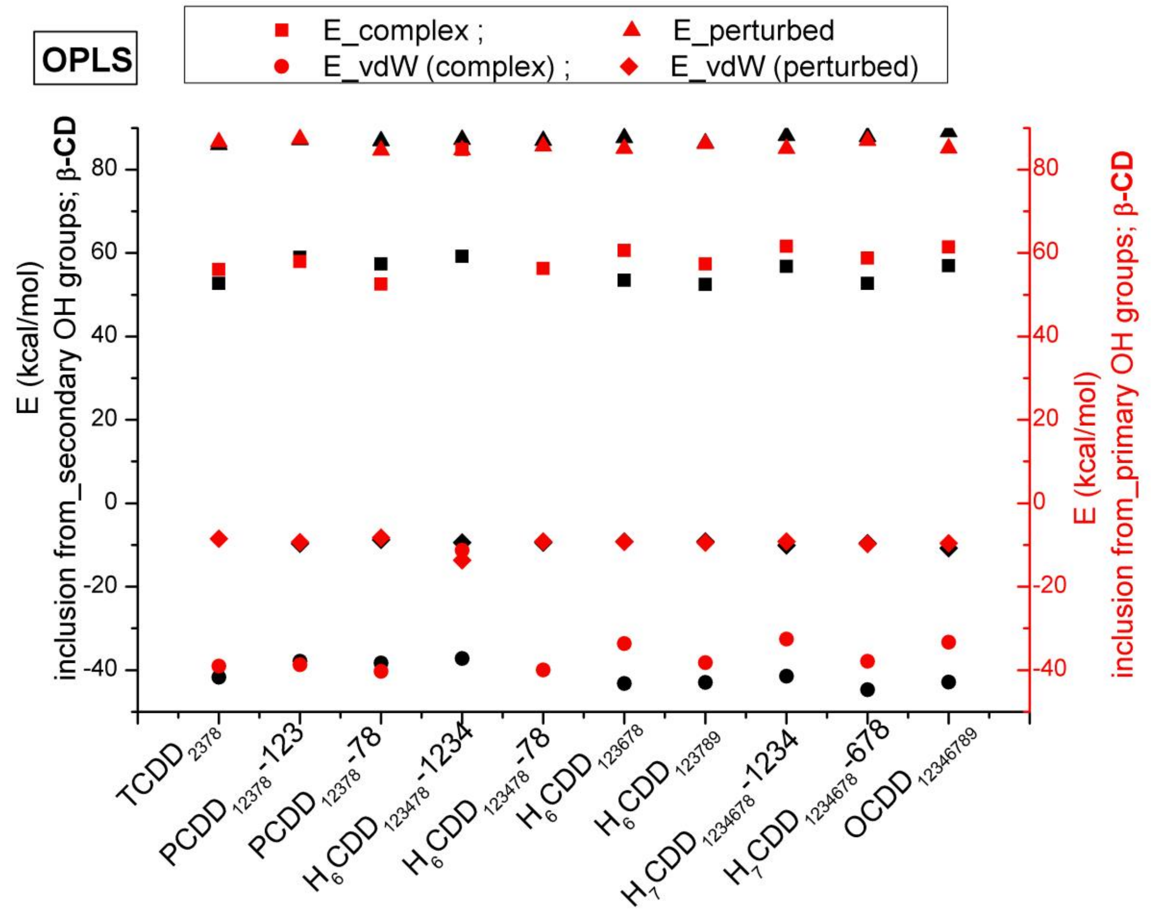

Figure S3. Complex and perturbed state energy (kcal/mol) and theirs vdW components for the inclusion complexes formed by the PCDDs approaching the secondary hydroxyl groups (black color) and the primary hydroxyl groups (red color) of β-CD by OPLS geometry optimization.

.

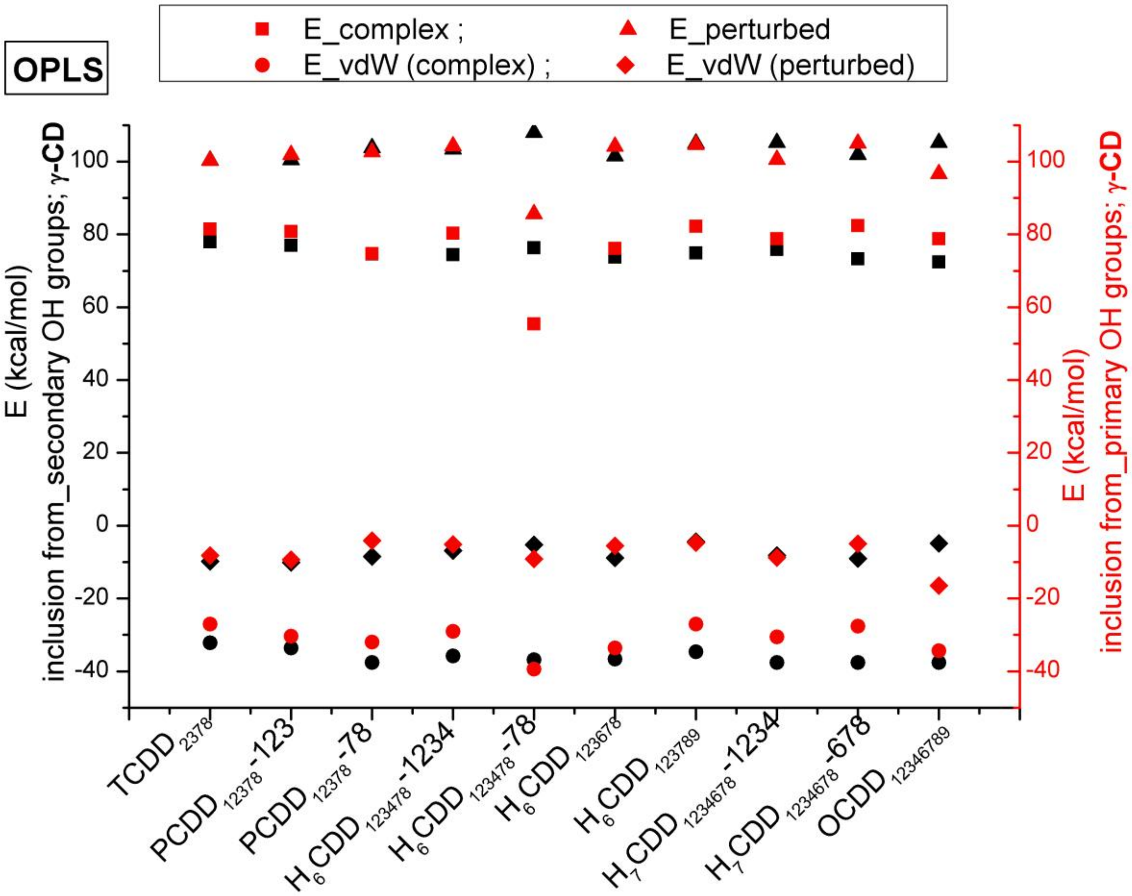

Figure S4. Complex and perturbed state energy (kcal/mol) and theirs vdW components for the inclusion complexes formed by the PCDDs approaching the secondary hydroxyl groups (black color) and the primary hydroxyl groups (red color) of γ-CD by MM+ geometry optimization.
